# The impact of testing and infection prevention and control strategies on within-hospital transmission dynamics of COVID-19 in English hospitals

**DOI:** 10.1098/rstb.2020.0268

**Published:** 2021-07-19

**Authors:** Stephanie Evans, Emily Agnew, Emilia Vynnycky, James Stimson, Alex Bhattacharya, Christopher Rooney, Ben Warne, Julie Robotham

**Affiliations:** ^1^ Modelling and Economics Unit, National Infection Service, Public Health England, London, UK; ^2^ Healthcare Associated Infection and Antimicrobial Resistance Division, National Infection Service, Public Health England, London, UK; ^3^ TB Modelling Group, TB Centre and Centre for Mathematical Modelling of Infectious Diseases, Faculty of Epidemiology and Population Health, London School of Hygiene and Tropical Medicine, London, UK; ^4^ Leeds Institute of Medical Research, University of Leeds, Leeds, UK; ^5^ Cambridge University Hospitals NHS Foundation Trust, Cambridge, UK; ^6^ NIHR Health Protection Research Unit in Healthcare Associated Infections and Antimicrobial Resistance at University of Oxford in partnership with Public Health England, Oxford, UK

**Keywords:** coronavirus, SARS-CoV-2, nosocomial transmission, mathematical model

## Abstract

Nosocomial transmission of SARS-CoV-2 is a key concern, and evaluating the effect of testing and infection prevention and control strategies is essential for guiding policy in this area. Using a within-hospital SEIR transition model of SARS-CoV-2 in a typical English hospital, we estimate that between 9 March 2020 and 17 July 2020 approximately 20% of infections in inpatients, and 73% of infections in healthcare workers (HCWs) were due to nosocomial transmission. Model results suggest that placing suspected COVID-19 patients in single rooms or bays has the potential to reduce hospital-acquired infections in patients by up to 35%. Periodic testing of HCWs has a smaller effect on the number of hospital-acquired COVID-19 cases in patients, but reduces infection in HCWs by as much as 37% and results in only a small proportion of staff absences (approx. 0.3% per day). This is considerably less than the 20–25% of staff that have been reported to be absent from work owing to suspected COVID-19 and self-isolation. Model-based evaluations of interventions, informed by data collected so far, can help to inform policy as the pandemic progresses and help prevent transmission in the vulnerable hospital population.

This article is part of the theme issue ‘Modelling that shaped the early COVID-19 pandemic response in the UK’.

## 1. Introduction

Nosocomial transmission of SARS-CoV-2, the transmission of the virus within a hospital, is a key concern in mitigating the spread of infection. The contribution of nosocomial transmission to the spread of COVID-19 in England is not yet known (as of February 2021), and there is variability around estimates of the risk of SARS-CoV-2 for healthcare workers (HCWs). Hospital inpatients represent an almost closed population where estimates of the disease latency can be used to predict the extent to which patients develop symptoms after hospital admission owing to progression of pre-symptomatic disease (community-acquired) versus those that become infected through nosocomial transmission. In HCWs, however, classifying the source of infection is difficult, as they work between wards and migrate between the hospital and community on a daily basis. Large European studies have estimated the overall proportion of HCWs infected with SARS-CoV-2 to range from 4% in Denmark (up to 23 April 2020) to 13.9% in Italy (up to 4 April 2020) [1,2]. English studies at an individual Trust level (a Trust being an organizational unit within the National Health Service, serving a defined geographical area) have estimated the prevalence of SARS-CoV-2 infection in HCWs in England to be between 15 and 45% for those in patient-facing roles, and 3 and 25% overall [3–6], with some studies citing occupational differences in infection risk [7], and others suggesting that infections in HCWs align with community incidence rates [8–10].

When contact patterns are known, genomic sequencing of pathogens can be used to build transmission trees to determine the origin and transmission chain of an outbreak [11,12]. One such study in an English hospital identified clusters of genomically linked cases between 13 March 2020 and 22 April 2020 involving 159 individuals, of which 124 (78%) were epidemiologically linked to other cases [13]. Outbreaks were identified on 12 wards, suggesting nosocomial transmission. HCWs accounted for 30/159 cases. However, these studies are resource-intensive and contact-tracing can be imperfect [14–16]. Mathematical models of infection transmission dynamics, when appropriately parameterized, rely on data that is widely collected and are a useful tool for estimating the source of infection in a hospital [17,18], and can subsequently be used to test hypotheses around testing and infection prevention and control (IPC) strategies on reducing transmission.

We present a within-hospital transmission model of SARS-CoV-2, including patients and HCWs, and use this model to quantify both the contribution of nosocomial infection to total infection burden within an English hospital and the effectiveness of alternative control measures.

## 2. Methods

### (a) Model description

**Figure 1.**
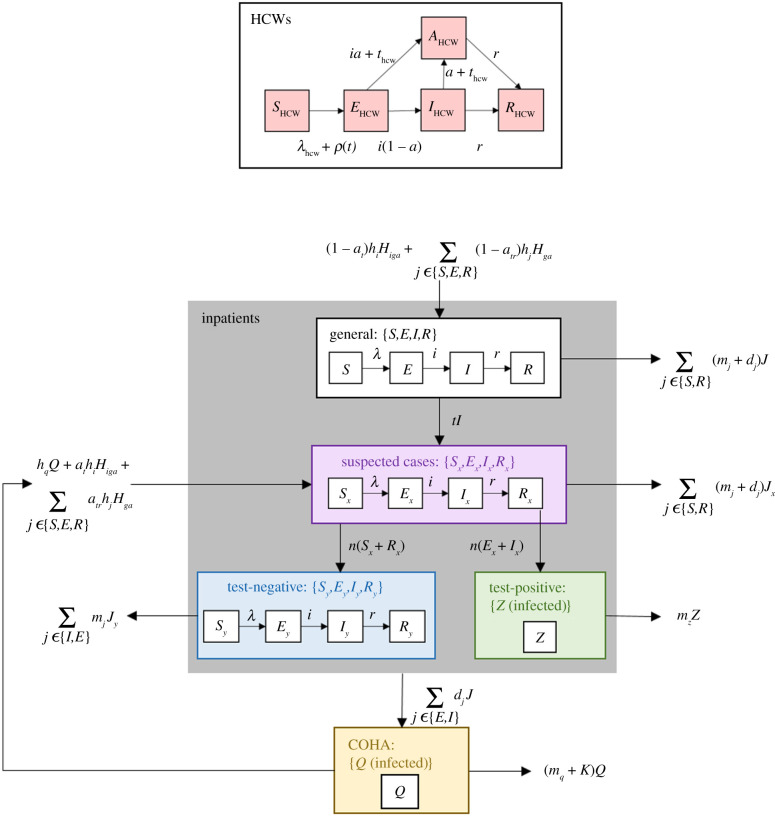
Schematic representation of the model. Parameter definitions and values, and model equations are given in the electronic supplementary material.

We present a susceptible–exposed–infected–recovered (SEIR) transmission model of COVID-19 infection within a hospital, with disease transmission to, from, and between both patients and hospital HCWs. The model includes testing of suspected cases on admission, and of symptomatic patients and HCWs within the hospital. Patients are placed into one of four distinct groups within the hospital: a general population for non-symptomatic admissions, and three test-dependent cohorts, namely, suspected cases, confirmed-positive cases and test-negative patients. Upon admission to the hospital, suspected COVID-19 patients are placed into the suspected cohort (awaiting test results) and subsequently move to either the positive or negative cohort upon receipt of test results. All admissions that are not tested are admitted to one of the remaining hospital beds, and undetected COVID-19 cases that enter the general hospital compartment can infect other patients and HCWs. HCWs can become infected either in the community, from infected patients in the hospital (including positives in cohorts) or from other infected HCWs. Figure 1 is a schematic representation of the model.

We use model simulations to examine the efficacy of HCW testing and patient isolation strategies.

Periodic testing of HCWs is a potential tool to prevent the spread of infections in the hospital. We consider periodic testing of the HCW population every 1, 7, 14 or 28 days, and assume that an equal proportion of the population are tested each day (so 1/7 of all HCWs will be tested every day for 7 day periodic testing, etc.). We assume that following a positive test result, infected and exposed HCWs are absent for 7 days.

We also explore two simulated scenarios for handling suspected cases as follows, with each impacting the transmission rates to and from patients:

*Scenario A (default)*: Suspected patients are cohorted together. Among those suspected cases are patients who are uninfected on admission and remain negative throughout, patients who are infected on admission and receive a positive test result, and those who were negative on admission but are exposed to infectious cases through being cohorted with those who test positive and then go on to become infected themselves. This last group of patients receive a negative admissions test but are infectious and can transmit to other patients in their cohort and also to HCWs. In this scenario, transmission can occur between patients in the testing cohort, as well as transmission to and from HCWs.
*Scenario B*: Suspected patients awaiting test results are all independently isolated using single rooms or bays within the hospital until test results confirm whether to move them into the tested-positive or tested-negative cohorts. To evaluate the effectiveness of single-room cohorting on IPC, we reduce the parameter for direct transmission between patients in the testing cohort by 25, 50, 75 and 100% in a scenario analysis. In all of these scenarios, we assume that it is feasible for the hospital to place all suspected patients in such single rooms or bays. Patient to HCW transmission is unchanged from Scenario A.

### (b) Assumptions

This model assumes there is no age, gender or race stratification within patients and HCWs. Patients cannot directly transmit the infection to other patients outside their own testing cohort, but beyond this, there is no separation of wards within the hospital. We define HCW to patient transmission to be transmission from an infected and infectious HCW to a susceptible patient, while patient to patient transmission represents transmission from an infected and infectious patient to a susceptible patient, noting however, that this may be mediated by (uninfected) HCWs acting as ‘vectors’. The model assumes full immunity of patients and HCWs once infected and therefore no individuals can be reinfected. We also assume that all exposed patients are infectious and asymptomatic (with the same infectiousness as that of symptomatic patients) and progress to the symptomatic state.

In the tested-positive cohort, patients are discharged on recovery. Within the suspected-positive cohort we assume patients will not be in the cohort long enough to recover from the infection and they are, therefore, discharged infected and infectious.

We assume that all patients and HCWs are susceptible to infection at the beginning of the epidemic and that test accuracy is 100%. We also assume that in the absence of testing 63% of HCWs continue to work while infected unless tested and found to be positive, as observed in a case study in The Netherlands [9].

### (c) Parameter estimation

The model is parameterized for an ‘average’ English hospital with 1000 beds and 8000 HCWs to reflect the average bed size to staff ratio in English NHS (NHSE) Trusts based on employee counts from a sample of trusts and bed numbers obtained from NHS records. Death rates and length of stay distributions are estimated using the R library *fitdistrplus* [19,20] and data from the Secondary Uses Service (SUS) COVID-19 dataset linked to laboratory data of positive tests from Public Health England's Second Generation Surveillance System (SGSS). The death probability is estimated by taking the mean of a polynomial fitted to the average probability of dying in hospital per year of age multiplied by the discharge probability from the SUS dataset, where the age distribution is sampled from the SUS dataset. For susceptible individuals, the death probability is calculated from the literature [21]. Data gathered from SUS comprises all completed hospital spells in NHSE Trusts arising from admissions over a nine-week period from 10 March 2020 to 11 May 2020 inclusive. Age (in years) and sex of the patient have been obtained, and length of stay is calculated to the nearest day using recorded admission and discharge dates. This amounts to a total of 119 000 positive admissions of patients who at some time (before the data extraction data on 29 June 2020) have tested positive for COVID-19, and 1.6 million negative admissions of patients who at no time (before 29 June 2020) have tested positive for COVID-19. Day cases, where the length of stay is zero, are excluded before deriving the distributions, leaving 93 000 positive admissions and 660 000 negative admissions. The length of stay distributions for positive and negative admissions are fitted using a Weibull distribution, adjusting for age and sex. Suitable values for transmission parameters between patients and HCWs are selected to quantitatively and qualitatively reproduce data from individual Trusts in different regions of England, with differential case-loads. To reflect the variability in case-loads and efficacy of infection control measures between Trusts, we include in the scenario and sensitivity analyses the impact of varying the rate of admission to hospital and transmission rates from zero to twice the calibrated values. This results in model runs that span the range of outcomes observed in the NHSE Situation Report (https://www.england.nhs.uk/statistics/statistical-work-areas/uec-sitrep/urgent-and-emergency-care-daily-situation-reports-2020-21/) data (electronic supplementary material, figure S1B).

The results presented are representative of regional averages and based on data from 9 March 2020 to 17 July 2020. The observed incidence rate in the community is likely an underestimate of the true incidence rate in the community population and therefore the results presented here could underestimate the contribution of community acquisition to infections in HCWs and also to the admissions rate of unidentified cases. However, policy changes advocating testing all patients on admission that were introduced at the end of April 2020 support the inclusion of only a small number of asymptomatic admissions, as in this study.

The full model code, differential equations, parameter values and data sources, can be found in electronic supplementary material.

### (d) Simulation

Simulations are initialized with a start date of 9 March 2020 and run for 130 simulated days, giving an end date of 17 July 2020. Admissions rates for symptomatically and asymptomatically infected patients are proportional to the regional average admissions rate per bed for the North East and Yorkshire region (taken to approximately represent a ‘medium’ incidence region). These are obtained by fitting a smoothing spline to the newly admitted cases data from NHSE Situation Report data from 23 March 2020 to 17 July 2020, and predicting dates earlier than 23 March using the spline and the ‘predict’ function in R (electronic supplementary material, figure S1A). Admissions rates of exposed patients that are not yet symptomatic are assumed to be 0.0025 multiplied by the admissions rate of symptomatic cases (i.e. for every 400 symptomatic patients that are admitted, one asymptomatic case is also admitted) based on the ratio of predicted prevalence in the population and hospital admissions. Population incidence rates (for community acquisition of infection by HCWs) are estimated by fitting a spline to raw data from the PHE Coronavirus tracker data (https://coronavirus.data.gov.uk/). It should be noted that the observed incidence rates are largely dependent on the availability of community testing and are, therefore, highly uncertain. To generate results that are generalizable to all regions, we explore high, medium and low incidence populations by multiplying the incidence rate and admissions rate by a factor of 2 (high), 1 (medium) and 0.5 (low) when generating our results (electronic supplementary material, figure S1A).

### (e) Uncertainty analyses

Uncertainty analyses are performed on parameters for which data are not available or are uncertain by generating 1000 random parameter samples and running the model with each parameter set. The parameters varied are: *a*, the probability a symptomatically infected HCW will self-isolate for 7 days without being tested; *b*_H2H_, *b*_H2P_, *b*_P2H_, *b*_P2P_, the scaling factor of the transmission rate (*β*) for transmission from HCWs to HCWs, HCWs to patients, patients to HCWs and patients to other patients, respectively; and *h*_i_, the scaling rate for the admissions rate of symptomatic cases (number of patients admitted per day = regional average × *h*_i_). Parameters are randomly sampled from a uniform distribution with a minimum of zero and a maximum of double the calibrated value. Under each parameter set, we simulate the nosocomial spread of SARS-CoV-2 from 9 March 2020 to 17 July 2020, randomly selecting admission rates from a single region in England for this analysis prior to the start of the simulation by drawing a region at random from the set of all English regions. Subsequently, partial-rank correlation coefficients (PRCC) are used to calculate the most significant parameter values using the *spartan* R package [22].

### (f) Model validation

We validate the model against patient data from the NHSE Situation Report and patient and HCW data from four individual NHS Trusts in England. The number of known cases in the simulations replicates the proportion of beds occupied by COVID-19 patients longitudinally (electronic supplementary material, figure S1B). To validate model results we compare against detailed data from an individual NHSE Trust (electronic supplementary material, figure S2), and for further validation of HCW results, we compare against PCR swabbing data from four NHSE Hospital Trusts (electronic supplementary material, figure S3), allowing for variability due to unknown detection rates in the Trust. Varying transmission rates and community incidence result in feasible dynamics of HCW infection.

### (g) Calculating the next-generation matrix and the within-hospital basic reproduction number

We use the approach of Heesterbeek and colleagues [23] to calculate the next-generation matrix (NGM) for transmission between hospitalized people *E*, those suspected to have infection (*E_x_*), those who test negative (*E_y_*) and HCWs (*E*_hcw_). This provides the reproduction numbers between different hospital population groups, given as the number of secondary infectious people generated by a typical infectious person in a given subgroup in the hospital population (for example, hospitalized patients) among people in another hospital population subgroup (for example, HCWs). The within-hospital basic reproduction number (*R*_0_) is calculated as the dominant eigenvalue of the NGM. The reproduction numbers is calculated using 1000 randomly sampled values for the unknown parameters (*a, h*_i_, *b*_P2P_, *b*_P2H_, *b*_H2P_, *b*_H2H_) and we calculate the resulting 95% range.

## 3. Results

### (a) Nosocomial transmission to patients and healthcare workers

**Figure 2.**
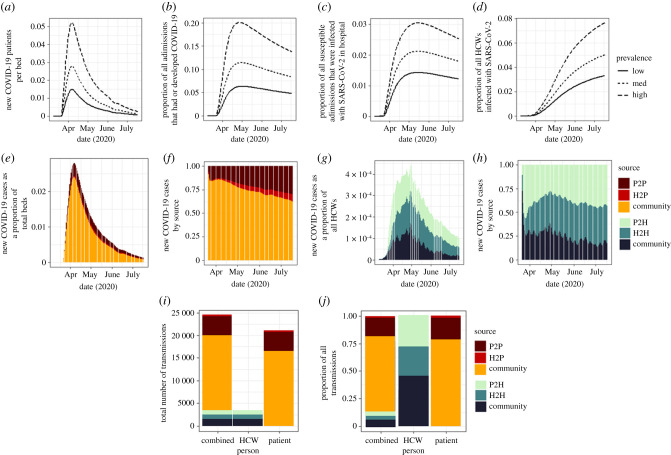
Sources of COVID-19 transmission in hospital. (*a*–*d*) Effect of population incidence rates on nosocomial transmission. (*a*) New COVID-19 positive patients per bed per day, (*b*) cumulative proportion of all admissions that are COVID-19 positive, (*c*) cumulative proportion of susceptible admissions that acquire COVID-19 in hospital, (*d*) cumulative proportion of HCWs that are infected with SARS-CoV-2. (*e*–*h*) Sources of infection in patients (*e*,*f*) and HCWs (*g*,*h*) for a setting with a medium population incidence rate. (*i*,*j*) Cumulative count (*i*) and proportion (*j*) of infections by source.

We simulated nosocomial infections in patients and HCWs at a typical English hospital from 9 March 2020 to 17 July 2020 under Scenario A with the assumptions that 37% of symptomatically infected HCWs are absent for 7 days following infection, and neither patients nor HCWs can be reinfected. We considered three different admissions rate/community incidence scaling factors: low, medium and high. Under a medium admissions rate/community incidence rate, new cases arise at a maximum rate of 0.0278 per bed per day at the peak of the epidemic, resulting in 11.43% of all patients being infected with SARS-CoV-2 over the simulation period (figure 2*a*,*b*). The vast majority (95.6%) of admitted patients are susceptible on admission, and of the 13 075 susceptible admissions under this setting, 243 (1.8%) acquire a nosocomial SARS-CoV-2 infection over the simulation period (figure 2*c*). The major source of infected patient cases of COVID-19 is admission of symptomatic cases from the community. However, the proportion of all patient cases of COVID-19 that are nosocomial in origin increases over time as the number of admissions from the community declines (figure 2*e*,*f*). Over the simulation period, the proportion of all patient cases that are nosocomial is 20.0%, and the primary route of nosocomial transmission to patients is from other patients—accounting for 84.6% of all nosocomial cases, and 16.6% of all cases (figure 2*i*,*j*).

Over the same time period, a total of 5.2% of all HCWs are infected (figure 2*d*), with a maximum of 33 HCWs infected at one time (0.41% of all staff, figure 2*g*), and a maximum of 18 HCWs absent owing to self-isolating with a true COVID-19 infection (0.21% of all HCWs). Unlike patients, in HCWs nosocomial transmission from both patients and other HCWs accounts for the majority of infections (37.1 and 36.2%, respectively, figure 2*i*,*j*), and the routes of the acquisition remain stable throughout (figure 2*h*). Although a higher number of patients have a nosocomial infection, proportionally the burden of nosocomial transmission is greater in the HCW population (figure 2*i*).

Scaling the admissions rate by a factor of 0.5 (low incidence) reduces the maximum rate of new cases to 0.0148 per bed per day (electronic supplementary material, figure S4A), and the total number of infected patients to 6.3% (figure 2*b*), 24.4% of which are nosocomial (1.1% of all susceptible admissions, electronic supplementary material, figure S4A, and figure 2*c*). When scaling by a factor of 2 (high incidence) these results are a maximum of 0.0518 new cases per bed per day, 20.1% of all patients infected and 16.2% of all infections caused by nosocomial transmission. The proportion of all admissions that acquired an infection in hospital is 2.9% (figure 2*a–c*). Under the same incidence scaling factors, the proportion of infected HCWs is 3.3% in a low incidence setting, 36.6% of which were nosocomial, and 7.7% in a high incidence setting, 65.2% of which are nosocomial (figure 2*c* and electronic supplementary material, figure S4A).

The number of secondary infections among patients resulting from a typical HCW is 0.00003, secondary cases in HCWs from a typical HCW is 0.217, secondary infections in HCWs from a typical patient is approximately 0.0001 (given that 91% of exposed asymptomatic patients are on a general ward), and in patients from a typical patient 0.44 (if suspected patients make up a small proportion of the population) (figure 2*e*). The overall within-hospital *R*_0_ is estimated to be 0.42 (95% CI 0.4, 0.45).

### (b) Efficacy of periodic testing of healthcare workers on nosocomial transmission events

**Figure 3.**
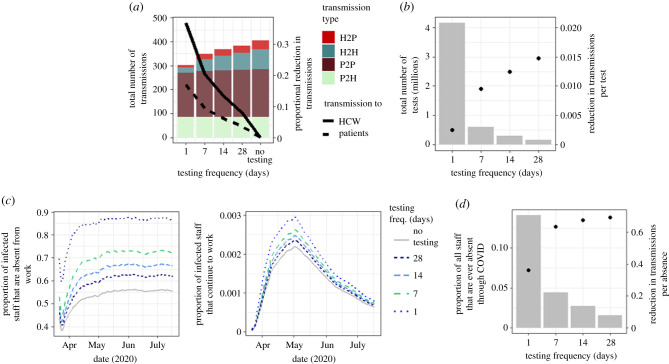
Effect of periodic testing of HCW on transmissions. (*a*) Cumulative number of transmissions by source (bars) and proportional reduction in transmissions to patients and HCWs (lines) when periodic testing is implemented from 9 March 2020 to 17 July 2020. (*b*) Total number of tests required under each testing scenario (bars) and overall reduction in transmissions per test (points) from 9 March 2020 to 17 July 2020. (*c*) Proportion of all infected staff that are absent from work under different testing scenarios (left), and the proportion of infected staff that continue to work each day (right). (*d*) Proportion of staff that are ever absent due to a COVID-19 infection (bars) and overall reduction in transmissions per absence (points) between 9 March 2020 and 17 July 2020.

Daily testing is the most effective at reducing transmission, with a reduction of 103 transmissions over the entire simulation period (25.4% of total transmissions) (figure 3*a*). The biggest proportional reduction is to HCWs, where there are 36.9% fewer transmissions overall, and 72.9% fewer HCW to HCW transmissions. There are 16.9% fewer transmissions to patients overall, and 72.5% fewer HCW to patient transmissions. Despite the relatively high efficacy in reducing transmissions, daily testing is highly inefficient, requiring over 4 million tests to be performed over the simulation period for a single hospital, resulting in an efficiency rate of 0.0001 transmission event reductions per test (figure 3*b*). The most efficient testing scenario is periodic testing every 28 days which required only 37 142 tests and reduces overall transmissions by 5.4% (22 transmissions total), transmissions to HCWs by 7.9%, and transmissions to patients by 3.6% generating an efficiency rate of 0.0006 transmissions prevented per test, six times higher than daily testing, but the efficacy (i.e. total number of transmissions prevented) is approximately 5 times lower. Testing every 7 days results in 57 fewer transmissions, 35 to HCWs (20.4% of total transmissions to HCWs), and 22 to patients (9.3% of total transmission to patients), required 148 571 tests, and has an efficiency rate of 0.0004, 1.8 times less effective than daily testing but 2.6 times more effective than testing every 28 days. Testing every 14 days results in 37 fewer transmissions, 23 to HCWs (13.4% of total transmission to HCWs), and 14 fewer transmissions to patients (6.1% of total transmissions to patients), required 74 285 tests, and has an efficiency rate of 0.0005. The 14 day periodic testing is 2.8 times less effective than daily testing, 1.8 times less effective than 7 day periodic testing and 1.7 times more effective than periodic testing every 28 days.

For low incidence scenarios, testing of HCWs has a similar effect, with a maximum reduction of 25.5% of all transmissions, but in a high incidence scenario, the maximum reduction of transmissions is 18.3% (electronic supplementary material, figure S5A). In a scenario where the probabilities of transmission events from HCWs are lower, daily testing of HCWs could still reduce overall transmissions to the HCW population by as much as 20%, and in a scenario where transmission from HCWs is twice as high (e.g. in the absence of adequate PPE), testing could reduce transmission to HCWs by up to 76%, and to patients by 49% (electronic supplementary material, figure S7).

Periodic testing of HCWs significantly reduces the proportion of HCWs who continue to work while infected. In a high, medium or low incidence setting, the maximum proportion of infectious HCWs that are self-isolating in a single day in the absence of testing is around 55%. When daily testing is introduced, 87% of infectious staff are off work, and for periodic testing every 7, 14 or 28 days this proportion is 72, 66 and 61%, respectively (figure 3*c* and electronic supplementary material, figure S5B). Testing does not significantly impact the proportion of staff absent from work with even daily testing only resulting in a maximum of 0.3% of all staff (24 individuals) absent from work in a single day (figure 3*c* and electronic supplementary material, figure S5B). The reduction of transmissions per absence is greatest when the testing rate is 28 days (i.e. when there are more infected staff) at an almost 70% reduction in transmission per absence, and lowest when the staff are tested daily (figure 3*d*).

### (c) Efficacy of single-room isolation versus cohorting of suspected cases on nosocomial transmission events

The majority of nosocomial infections under Scenario A (suspected COVID-19 positive patients cohorted together) are caused by (direct or indirect) patient to patient transmission (figure 2*i*,*j*). This suggests that improving IPC mechanisms within hospitals would be effective in lowering the rate of nosocomial infections in the inpatient population, which would subsequently reduce the number of HCWs infected.

**Figure 4.**
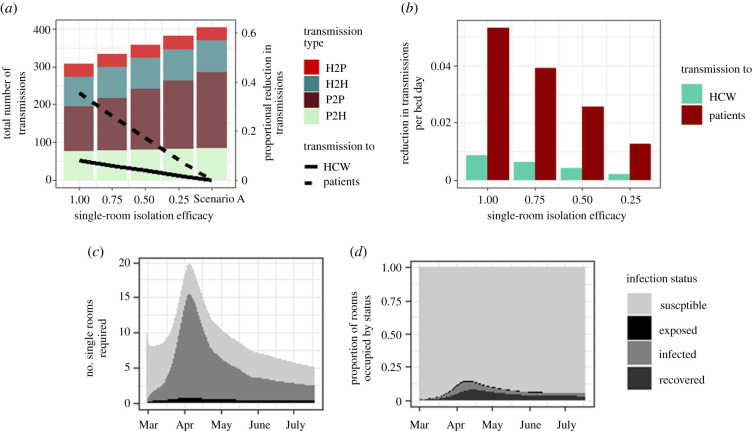
Effect of isolating suspected cases in single rooms versus cohorts. (*a*) Number of transmissions (bars) and proportional reduction in transmissions (lines) when patients are isolated in single rooms and transmission rates are reduced by various amounts. (*b*) Reduction in transmissions to patients and HCWs per single bed day over the entire simulation period. (*c*) Number of single rooms occupied per day by patients undergoing testing for COVID-19 who are susceptible, infected or exposed. (*d*) Proportion of rooms occupied by susceptible, exposed, infected and recovered patients over time.

To explore this hypothesis, we simulated a situation where instead of cohorting suspected-positive patients together, suspected patients are isolated in single rooms or bays within a hospital while they wait for their test results to come back, taking on average 1.18 days (electronic supplementary material, table S1). In a medium incidence setting, there are fewer nosocomial infections under Scenario B (placing suspected cases in single rooms/bays) than Scenario A (group cohorting of suspected cases) under different levels of transmission reduction (figure 4*a*). The reduction in transmissions increases linearly with efficacy, with 24 fewer transmissions when the probability of transmission is reduced by 25% (5.9% of all transmissions under Scenario A) to 97 fewer transmissions when transmissions from patients within the same cohort is reduced by 100% (24.1% of all transmissions under Scenario A), with the largest effect on transmissions to patients (8.2–35.3% reduction compared with Scenario A). The number of single-room or bay bed days required over the entire simulation period under Scenario B is approximately 1564, and the reduction in transmissions per single isolation bed day used ranges from 0.015 to 0.062 (figure 4*b*), with the majority of reductions coming from patient to patient transmissions. The maximum number of single rooms or bays required at any one time is 28 under a medium setting (49 for a hospital in a high incidence setting) (figure 4*c* and electronic supplementary material, figure S6C). At the peak of the epidemic, the majority of these rooms are occupied by true cases, but as the community incidence declines a higher proportion of single rooms are occupied by susceptible individuals (15.4% versus 74.7%). These trends are echoed in the low and high incidence settings (electronic supplementary material, figure S6), and also under scenarios where transmission rates from patients are scaled up or down by a factor of 2 (electronic supplementary material, figure S7).

### (d) Uncertainty and sensitivity analyses

We performed an uncertainty analysis to determine the effect of variability around parameters related to admission of infected cases, transmission and staff isolation rates as described in Methods (electronic supplementary material, figure S8). Under this range of parameter values, the temporal dynamics of known COVID-19 cases in all hospitals can be captured (electronic supplementary material, figure S1B). We demonstrate that up to 20.3% of all beds in a Trust are filled with known COVID-19 cases at the peak of the epidemic (mean 5.7, s.d. 3.8, electronic supplementary material, figure S1A). Overall, the mean proportion of all cases that are nosocomial under these parameter sets is 17.4% (s.d.13.4), with a maximum of 2% of all patients that are susceptible on admission developing an infection in hospital at the peak of the epidemic. In a medium incidence scenario, however, the mean proportion of patients that develop an infection in hospital is less than 1% (mean 0.93, s.d. 0.72; electronic supplementary material, figure S1B).

To assess the sensitivity of the number of patient and HCW transmissions that are nosocomial in origin to the parameters of interest, we calculated PRCCs temporally (electronic supplementary material, figure S8C). Early in the simulation when community admission rates are high, patient to patient transmission rates drive the number of nosocomial infections in the patient population and this is demonstrated by high PRCCs (0.625–0.75) for the parameter *b*_P2P_ at early timepoints. However, as the admission rates drop there is an increasing role for HCW to patient transmission, indicated by the increasing PRCCs for parameters *b*_H2H_ and *b*_H2P_ at later timepoints. The number of nosocomial transmissions in the patient population is strongly influenced by the admission rate (*h*_i_) through the entire simulation. The self-isolation rate of symptomatic HCWs (*a*) has a minor, non-significant effect on the number of nosocomial infections in patients. For HCWs, patient to HCW transmission rates and the admission rate of infected patients are influential throughout the simulated time period, and there is an increasingly important role for HCW to HCW transmission at later time points.

## 4. Discussion

Using an SEIR model of transmission, we studied the potential sources of SARS-CoV2 infections in a simulated ‘average’ English hospital with 1000 beds and 8000 HCWs. Our results suggest that while the majority of cases in hospitalized patients are a result of community acquisition of the virus, direct and indirect patient to patient transmission drives nosocomial infection. Furthermore, the majority of cases in HCWs were not found to be community-acquired, but instead driven by nosocomial transmission. This is in contrast to an earlier study that suggested that the rate of asymptomatic infection among HCWs more likely reflects general community transmission than in-hospital exposure [24], although the proportion of infected cases observed by other studies such as Treibel *et al.* [24] is in line with estimates presented in this study. More detailed genomic epidemiology studies are required to disentangle the source of transmissions to HCWs. Through the NGM approach of [23], we estimated that the within-hospital *R*_0_ is 0.45, suggesting that the epidemic within hospitals is not self-sustaining and would die out within four generations without infectious admissions. Both patients and other HCWs play an important role in HCW infections (figure 2).

Given that HCW to HCW infections were identified as a primary contributor to nosocomial infections in the HCW population, we evaluated the potential for periodic testing to reduce infection. In our simulated hospital, we considered a scenario where the entire workforce is tested every 1, 7 or 14 days, and a positive HCW result leading to isolation at home for 7 days from the date of the test (figure 3). We observed only a small proportion of HCWs absent at any one time (up to 0.3%) owing to testing positive, which is considerably less than current observations, which are in the region of 25% of staff absent owing to suspected COVID-19 infection or self-isolation (NHSE Situation Reports). This would, therefore, suggest that testing HCWs routinely is unlikely to cause an unsustainable decline in the number of available workers. There is also a trade-off between reduction in transmission and efficiency, with the most efficient testing scenario only 20% as effective as the least efficient.

We suggest that HCW testing will have a moderate effect on patients (up to 16.9% fewer infections from HCWs) but a larger effect on HCWs (up to 72.5% fewer HCW to HCW transmissions, and 36.9% fewer HCW infections in total). This is supported by findings by Grassley *et al*. [25], who suggest that regular screening can prevent transmissions in hospitals and other care facilities, and Chin *et al.* [26], who suggest that lower-frequency HCW testing is ineffective in reducing transmission.

We find IPC measures, specifically patient isolation, in hospitals to have a larger effect of up to a 72% reduction in nosocomial transmission events to HCWs (figure 4).

The management of suspected cases on admission to hospital has the potential to significantly reduce the rate of nosocomial infection. Our model suggests that managing symptomatic patients in single rooms or bays that are fully disinfected in between patients could reduce nosocomial infection rates by up to 24.1%, with a 35.3% reduction in transmissions to patients. If only symptomatic patients are isolated, a maximum of 2.7% of all beds are required to be single rooms in a medium incidence setting when tests have a turnaround time of approximately 28 h; however, in a higher incidence setting this could be as high as 4.8%. If the turnaround time for testing was reduced, the number of beds required, and the number of nosocomial infections, would fall. In a scenario where a higher number of patients had illnesses, such as influenza, that could result in them wrongly suspected of having COVID-19 owing to the similarity of symptoms, a higher number of beds would be required. In practice, as the criteria for patients to be tested change to include larger cohorts of patients, it may not be possible to use single rooms or bays, and in this scenario cohorting patients by suspected infection status would become the most practical option for preventing transmission. Controlling and minimizing the risk of nosocomial transmission can limit the development of further infections and their subsequent effects on community transmission [27,28]. These results have important implications when considering the design of temporary facilities such as the Nightingale Hospitals [29]. While the model is parameterized using the best data currently available, data on contact patterns (of different groups) of HCWs with COVID-19 patients would improve parameterization and increase certainty in outputs. Simulations with increased transmission rates from patients and HCWs give qualitatively similar results to what we have observed here but the values change depending on the exact parameterization (electronic supplementary material, figure S7). A limitation of this work is that this model does not separate asymptomatic from pre-symptomatic patients. Also, the assumption that asymptomatic individuals are equally as infectious as symptomatic individuals, and the absence of age, gender or race-specific risk factors may not reflect reality, and as evidence develops such parameters can be updated. Further, the model is deterministic and cannot account for the impact of outbreaks on individual wards, or the sequestering of patients and HCWs onto ‘hot’ and ‘cold’ wards. Our results are based on average admissions rates for every hospital in a particular region (number of admissions per hospital bed); this does not account for Trusts that are specialists in certain areas and therefore receive a larger proportion of COVID-19 cases than other Trusts in the same region. We have assumed that the risk to HCWs in the community is related only to community incidence and not increased by them being unable to work from home or the need for HCWs to use public transport in certain areas (e.g. London). We acknowledge that the observed incidence rate in the community is likely an underestimate of the true incidence rate in the community population as a whole. Further, little is known about the transmission dynamics between HCWs and individuals in the community during this pandemic and it is, therefore, possible that the contribution of community acquisition to the number of infected HCWs has been underestimated. In the case where the community acquisition rate of SARS-CoV-2 by HCWs is higher, we would expect that our estimated transmission probability from HCWs to other HCWs (*b*_H2H_), and therefore the number of transmissions from HCW to HCW would be reduced to maintain calibration with observed data. We have assumed a 100% accuracy rate of PCR testing for identifying COVID-19 cases. The accuracy of testing and in particular the presence of false negatives in the true-negative cohort would increase the risk of transmission between patients, and to patients and HCWs; however, we would not expect the qualitative behaviours exhibited by the model, or the results of altering transmission rates to change significantly under reasonable uncertainty around the accuracy of the PCR tests.

Epidemic models in which most of the parameters are constant are a useful tool for evaluating the potential impact of interventions over a fixed setting. In reality, guidance on best practice changes regularly, and therefore to measure the true impact of measures to reduce the spread of COVID-19 inside hospitals, certain parameters should be time-varying to reflect the changing advice and guidance. This work focuses on a setting where the protocol remains constant from baseline, giving an upper bound on the estimate of efficacy for each scenario, and the results should be considered in this context. The temporal dynamics in this study are fixed to those from the North East and Yorkshire region (a medium incidence area); thus there may be a shift in the timescale of events in regions that were affected earlier, such as London and the West Midlands. This study demonstrated a marked reduction in nosocomial transmission events through the use of HCW screening and prompt isolation of suspected cases. The model was calibrated to data from English Trusts during a time period when face coverings were also introduced universally, and this preventive measure is reflected in the transmission probabilities. Over the course of the first wave of the pandemic, a number of other interventions were introduced in the UK, ranging from more extensive use of personal-protective equipment to rapid diagnostic testing. In combination, these measures are likely to further reduce hospital-associated transmission of SARS-CoV-2 in subsequent phases of the pandemic.

Despite these limitations, the results from this work have the potential to impact infection control during subsequent coronavirus outbreaks and help inform policy intending to reduce the number of nosocomial infections.

## In context

The model presented in this paper was developed to assess the impact of strategies for cohorting patients by COVID-19 status on admission to hospital, and to provide estimates on the efficacy of testing strategies for reducing the risk of nosocomial transmission in England. Findings, particularly those highlighting the potential importance of healthcare worker (HCW) to HCW transmission and impact of regular testing, helped to inform policy decisions aiming to prevent infection in HCWs and transmission in the hospital setting (e.g. https://www.sehd.scot.nhs.uk/dl/DL(2020)29.pdf). The analysis was performed as the UK neared the end of the first-wave of SARS-CoV-2 infections, but in the other work outside of the scope of this paper, the model has been able to adequately capture trends in nosocomial infections for different settings with differing rates of hospital admissions of COVID-19 cases.

Several key outputs of this work have since been observed in other studies. An independent study from Public Health England estimated the risk over the entire first wave to be approximately 1% [30]. In this work, we determined that the risk of an individual patient contracting a nosocomial SARS-CoV-2 infection was in the range of 1–3% meaning 0.6–2.5% would be symptomatic and therefore likely to be detected. We also demonstrated that only high-frequency HCW testing was efficacious at reducing transmissions within the HCW population, a result that was echoed by other modelling studies [25,26]. A study by the Health and Safety Investigation Branch identified the potential for HCW to HCW transmission to occur while in the hospital, supporting our modelling results which suggested that over the first wave, HCW to HCW transmission was responsible for approximately a one-third of the risk to an HCW [31]. Further, the REACT-2 study determined that HCWs were twice as likely to test positive for SARS-CoV-2 antibodies as the general population [32], indicating that the risk to HCWs on shift should be greater than that in the general community. This result was also reflected in our model parameterizsation.

We highlighted several limitations in this work including the lack of a distinction between asymptomatic and pre-symptomatic patients, the assumption of 100% accuracy of PCR -testing and that patients are not sequestered into wards based on their infection status. Through further model developments, we are addressing these issues to allow more complex experimentation, including greater insight into routes of transmission within the hospital setting, alternative patient testing strategies and implications of test characteristics. Current indications are that results from the updated and extended models echo those presented in this work.

## Data accessibility

Full model code and parameter values can be found in electronic supplementary material, File. Population incidence rates are available from https://coronavirus.data.gov.uk/. Data from individual NHSE trusts are not publicly available.
